# Changes in Inflammatory Markers Following Bariatric Surgery and the Impact of the Surgical Procedure: A 12-Month Longitudinal Study

**DOI:** 10.1007/s11695-024-07629-z

**Published:** 2025-05-27

**Authors:** Rasmus Tanderup Jensen, Anne Cathrine Baun Thuesen, Yun Huang, Sara Elizabeth Stinson, Helene Bæk Juel, Sten Madsbad, Flemming Bendtsen, Torben Hansen, Julie Steen Pedersen

**Affiliations:** 1 Novo Nordisk Foundation Center for Basic Metabolic Research, Faculty of Health and Medical Sciences, University of Copenhagen, Copenhagen, Denmark; 2 Section for Precision Psychiatry, Division of Mental Health and Addiction, Oslo University Hospital, Oslo, Norway; 3 Department of Endocrinology, Copenhagen University Hospital Hvidovre, Copenhagen, Denmark; 4 Gastrounit, Medical Section, Copenhagen University Hospital - Amager and Hvidovre, Hvidovre, Denmark, Dept of Clinical Medicine, University of Copenhagen, Copenhagen, Denmark

## Abstract

**Background:**

Obesity is associated with an increased risk of cardiometabolic morbidity and mortality, which may be attributable to systemic low-grade inflammation. The impact of bariatric surgery-induced weight loss on low-grade inflammation has not yet thoroughly been described. We investigated the effect of Roux-en-Y gastric bypass (RYGB) and sleeve gastrectomy (SG) on the plasma levels of cytokines, chemokines, and cytokine receptors prior to surgery (baseline), and then three and 12 months after surgery.

**Methods:**

We recruited 68 individuals (41 females, 27 males) with severe obesity (42.84 ± 6.28) who had been referred for bariatric surgery (RYGB: *n* = 29, SG: *n* = 39). Blood samples were collected after an overnight fast at baseline (immediately before surgery), 3 and 12 months after surgery. Eleven patients without obesity or cardiometabolic disease served as controls at baseline. Ninety-two plasma proteins were measured using an Olink Target 96 inflammation panel.

**Results:**

We used a linear mixed model to test differences in inflammatory markers at baseline, across time points and between groups. At baseline, 36 cytokines were found to be differentially expressed between the bariatric surgery patients and controls. Of these cytokines, 13 had significantly decreased three months after bariatric surgery and 27 had significantly decreased 12 months after surgery, compared with baseline. Two cytokines (CCL25 and CCL28) increased markedly after 12 months. Only one cytokine (CCL25) was significantly different between the procedures performed, where it increased in the RYGB group 12 months after surgery.

**Conclusion:**

Individuals with severe obesity have increased expression of plasma inflammatory cytokines compared to controls, but low-grade inflammation improves following bariatric surgery, regardless of whether it is RYGB or SG.

**Supplementary Information:**

The online version contains supplementary material available at 10.1007/s11695-024-07629-z.

## Introduction

Excess weight and obesity affect an estimated 60% of the population in Europe and are a serious public health concern [1]. Obesity induces metabolic dysfunction (MD) and increases the risk of cardiometabolic diseases, including type 2 diabetes (T2D), metabolic dysfunction-associated steatotic liver disease (MASLD), and cancer, all of which are leading causes of global morbidity and mortality [2]. The metabolic dysfunction in obesity is linked to the presence of systemic low-grade inflammation, which primarily originates from the presence of inflamed adipose tissue [3–5].

Adipose tissue is believed to be an important regulator of inflammatory cytokine secretion. During weight gain, the adipose tissue phenotype is transformed due to infiltration of immune cells into the adipose tissue, the emergence of dysfunctional adipocytes which result in inflammation of the adipose tissue [6–8]. The subsequent release of proinflammatory cytokines has systemic effects [7, 9]. Thus, the reversal of inflammation has been proposed as a treatment target for MD.

Several studies have investigated whether bariatric surgery can reverse or improve obesity-associated, chronic, low-grade inflammation. Overall, bariatric surgery seems to improve the cytokine profile by lowering chronically elevated cytokine levels [10–13], such as interleukin-6 (IL-6), but to our knowledge only one study has comprehensively investigated the cytokine profile of 76 markers in obesity and the effects of bariatric surgery [14]. However, this study by Wolf et al. [14] only included 25 patients undergoing bariatric surgery (RYGB and SG), with follow-up at 3 and 6 months post-surgery, and it did not report on the differences in inflammatory markers and their association with glucose metabolism, nor on any differences found between surgery types. Owing to the considerable differences that exists between the RYGB and SG surgical procedures, it is important to investigate and compare the subsequent effects on plasma inflammation.

In this study, we perform a thorough characterization of plasma cytokines, chemokines, and cytokine receptors using state-of-the art proximity extension assays among individuals with obesity undergoing RYGB or SG, with follow-up 3 months and 12 months after surgery. Furthermore, we studied the differential impacts of the two surgical procedures on the inflammatory profile post-surgery. We hypothesized to find significant variances in the changes in the cytokine patterns following the different types of surgery: RYGB vs SG.

## Materials and Methods

### Participants

This study included 68 individuals with obesity who were referred to Copenhagen University Hospital, Hvidovre for bariatric surgery (RYGB: *n* = 29, SG: *n* = 39) between November 2016 and September 2019.

In Denmark, patients undergoing bariatric surgery must meet the criteria for bariatric surgery, which are issued by the Danish Health Authorities. Consequently, the same criteria applied to be enrolled in the present study. During 2016–2019, these criteria were an age between 25 and 70, a body mass index (BMI) > 35 kg/m^2^ and at least one of the following obesity-related disorders; dyslipidemia, T2D, hypertension, sleep apnea, polycystic ovarian syndrome, and/or arthrosis in the lower extremities; or a BMI > 50 kg/m^2^ with/without obesity-related comorbidities. There were no additional study specific inclusion criteria. As we in this patient cohort studied, the prevalence of MASLD and severity of low-grade inflammation, study specific exclusion criteria were preexisting liver disease other than MASLD, clinical signs of infection at inclusion and intake of antibiotics of any kind prior to one month before inclusion in the study.

This study included three patient visits: baseline (the morning of the day of surgery), three, and 12 months after surgery. Sixty-eight study subjects were available for the baseline analyses and 40 study subjects were available for the analyses at three and 12 months after surgery.

Because we in this study cohort also investigated the effects of surgery on MASLD and liver fibrosis [15] liver tissue was sampled during surgery but it was optional to undergo a percutaneous re-biopsy of the liver at 12 months. Forty patients accepted renewed sampling of liver tissue 12 months after surgery, and consequently, these 40 study subjects were also available for the present study. At baseline, there were no significant differences in clinical, biochemical or anthropometrical measures between the 40 subjects available for 12 months analyses and the 28 subjects only available for analyses at baseline.

In addition, we enrolled a control group of 11 healthy patients without obesity. The control subjects were taking no medications other than oral contraceptives, occasional painkillers, and/or anti-allergy medicine. Clinical and anthropometric data and fasting blood samples from the controls were collected once, on the morning of the day of the cholecystectomy.

### Ethics

The study was approved by the Scientific Ethics Committee of the Capital Region of Denmark. All participants gave written informed consent for their participation. The study conforms to the Declaration of Helsinki.

### Anthropometric and Standard Biochemical Measurements

Clinical and anthropometric data, as well as fasting blood samples, were collected at each study visit. Standard biochemical analyses were run immediately after blood sampling, as described elsewhere [16]. The blood designated for later analysis of plasma inflammatory markers was immediately centrifuged to retrieve the plasma, which was then instantly pipetted and stored in the research biobank at − 80 °C. The Olink analyses was conducted in 2021.

The homeostasis model assessment of insulin resistance (HOMA-IR) was calculated according to the formula (fasting glucose [mg/dL] × insulin [mU/L]/405) [17].

### Analysis of Inflammatory Markers in Plasma

Cytokine expression in plasma was measured using the Target 96 inflammation assay (Olink Proteomics AB, Uppsala, Sweden). Raw normal protein expression levels (NPX) values were bridged and normalized to 16 control samples using the OlinkAnalyze R package (https://cran.r-project.org/web/packages/OlinkAnalyze/index.html). Cytokines were excluded if more than 75% of samples were below the limit of detection or more than 75% of sample measurements were missing. After filtering and quality control, 18 cytokines were excluded, leaving 74 cytokines for analysis. Inverse transformation was applied to all cytokine data to ensure that model assumptions were met. A total of 68 individuals, and all 11 controls, had measures available at baseline and 40 individuals (out of initial 68 individuals) did after three months and 12 months.

### Statistical Analyses

All data were checked for normal distribution using histogram plots and Shapiro–Wilk test. Non-normally distributed data were log-transformed. Non-normally distributed data after log-transformation are given as medians and interquartile ranges in the tables, while normally distributed data are given as means and standard deviations.

A two-way analysis of variance (ANOVA) and Kruskal–Wallis test were used to test for significant differences between the three groups at baseline (RYGB vs. SG vs. controls) (Table 1). RYGB and SG groups were combined for the analyses across the three time points (baseline, 3 months post-surgery, and 12 months post-surgery). Biochemical and anthropometric data were also analyzed using a two-way ANOVA to investigate changes after bariatric surgery compared to baseline (Supplementary Table 1). To test the differences in biochemical and anthropometric measures in each group across the three time points, a Tukey HSD test and a pairwise Wilcoxon test were used (Table 2). Fisher’s exact test was used to test for differences in categorical variables between the two surgery groups (Table 2).

**Table 1:** Baseline clinical characteristics in the three study groups at baseline

	SG	RYGB	Controls	*p*-value
Baseline	*n* = 39	*n* = 29	*n* = 11	
Age (years)	44.46 ± 9.9	44.28 ± 8.32	39.73 ± 7.9	0.43
Sex (F/M)	23/16	18/11	8/3	0.77
Height (m)	1.73 (0.15)	1.73 (0.21)	1.71 (0.13)	0.72
Weight (kg)	127.25 ± 21.04	130.11 ± 25.05	73.43 ± 12.75	1.99e-10*
BMI (kg/m^2^)	42.24 ± 5.5	43.68 ± 7.3	24.96 ± 2.45	3.13e-13*
Waist circumference (cm)	122.17 ± 14.71	123.9 ± 12.3	85.36 ± 9.05	3.07e-12*
Hip circumference (cm)	132 (20)	135 (23)	99 (8.5)	9.29e-07*
WHR	0.94 (0.19)	0.89 (0.11)	0.85 (0.14)	0.15
Glucose (mmol/L)	6 (1.1)	6.1 (1)	5.6 (0.7)	0.05*
Insulin (pmol/L)	134 (80.5)	118 (81)	64 (48)	0.003*
C-peptide (pmol/L)	1210 (380)	1220 (444)	863 (271)	0.0004*
HbA1c (mmol/mol)	37.37 ± 5.65	35.9 ± 4.64	32.18 ± 3.49	0.018*
HOMA-IR	5.73 (3.9)	4.68 (2.84)	2.13 (3.93)	0.002*
hsCRP (mg/L)	3.8 (5.35)	5.65 (5.2)	0.9 (0.68)	2.47e-04*
Total cholesterol (mmol/L)	4.34 ± 0.96	4.32 ± 0.82	4.64 ± 0.84	0.59
LDL (mmol/L)	2.5 (1.1)	2.4 (0.6)	2.8 (0.85)	0.24
VLDL (mmol/L)	0.6 (0.3)	0.6 (0.5)	0.5 (0.3)	0.26
HDL (mmol/L)	1.18 ± 0.31	1.18 ± 0.3	1.34 ± 0.24	0.28
Triglycerides (mmol/L)	1.24 (0.65)	1.28 (0.96)	1.1 (0.58)	0.27

Data are represented as either mean ± SD or median (IQR), * indicates a *p*-value < 0.05 and significance was tested with a two-way ANOVA, Kruskal–Wallis, or Fisher’s exact test. Body mass index (BMI), waist-hip ratio (WHR), low-density lipoprotein (LDL), very low-density lipoprotein (VLDL), high-density lipoprotein, high-sensitivity C-reactive protein (hsCRP)

**Table 2:** Anthropometric and biochemical measures at baseline, 3 months, and 12 months after surgery in SG and RYGB groups

	SG					RYGB				
	Baseline	3 months	*p*-value (BL-3 M)	12 months	*p*-value (BL-12 M)	Baseline	3 months	*p*-value (BL-3 M)	12 months	*p*-value (BL-12 M)
*n*	*n* = 16	*n* = 16		*n* = 16		*n* = 24	*n* = 24		*n* = 24	
Weight (kg)	123.0 ± 16.83	105.4 ± 14.79	0.0007*	94.57 ± 14.84	0.0001*	129.6 ± 23.06	111.4 ± 20.7	0.051	95.07 ± 17.22	0.0001*
BMI (kg/m^2^)	41.00 ± 4.75	35.23 ± 4.4	0.0001*	31.48 ± 4.0	0.0001 *	43.43 ± 7.23	37.4 ± 6.74	0.047 *	32.00 ± 6.13	0.0001 *
Waist circumference (cm)	118.2 ± 10.53	103.5 ± 9.7	0.0001 *	96.46 ± 12.0	0.0001*	125.3 ± 11.58	110.7 ± 14.18	0.01 *	99.7 ± 13.16	0.0001 *
Hip circumference (cm)	129 (23)	119 (20)	0.04*	112 (19)	0.0002*	134 (18)	120 (15)	0.098	107 (14)	0.002*
WHR	0.89 (0.11)	0.88 (0.17)	0.57	0.86 (0.19)	1	0.88 (0.11)	0.89 (0.15)	1	0.85 (0.1)	0.35*
Glucose (mmol/L)	6.3 (1.05)	5.4 (0.5)	0.0002*	5.35 (0.4)	0.0002*	6.3 (1)	5.5 (0.8)	0.15	5.4 (0.5)	0.04*
Insulin (pmol/L)	106 (52)	73 (57)	0.0001*	63 (39)	0.001*	106 (52)	55 (41)	0.02*	51 (20)	0.001*
C-peptide (pmol/L)	1220 (357)	914 (316)	0.005*	811 (302)	0.0005*	1220 (357)	827 (348)	0.0002*	717 (220)	0.03*
HbA1c (mmol/mol)	36.9 ± 5.49	38.45 ± 14.56	0.94	36.9 ± 13.62	0.99	36.9 ± 5.49	37.08 ± 7.37	0.99	35.27 ± 5.44	0.82
HOMA-IR	4.78 (1.93)	2.42 (1.85)	2.8e-05*	2.12 (1.18)	3.2e-05*	4.8 (1.93)	2.23 (1.58)	2.1e-05*	1.68 (0.55)	0.0009*
hsCRP (mg/L)	6.4 (5.3)	2.5 (3.8)	0.5*	0.85 (1.3)	7.6e-05*	6.4 (5.3)	2.3 (3.4)	0.15	0.75 (0.83)	0.004*
Total cholesterol (mmol/L)	4.56 ± 1.04	4.32 ± 0.96	0.76	4.54 ± 1.42	0.99	4.05 ± 0.43	3.34 ± 0.5	0.0003*	3.35 ± 0.4	0.0003*
LDL (mmol/L)	2.3 (0.5)	2.6 (0.9)	1	2.65 (1.45)	1	2.3 (0.5)	1.75 (0.48)	0.045*	1.7 (0.35)	0.001*
VLDL (mmol/L)	0.6 (0.55)	0.4 (0.2)	0.08	0.4 (0.15)	0.04*	0.6 (0.6)	0.4 (0.1)	0.14	0.3 (0.1)	0.0003*
HDL (mmol/L)	1.22 ± 0.33	1.25 ± 0.28	0.94	1.52 ± 0.34	0.005*	1.11 ± 0.23	1.09 ± 0.22	0.99	1.41 ± 0.28	0.032*
Triglycerides (mmol/L)	1.28 (1.13)	0.98 (0.38)	0.15	0.9 (0.39)	0.06	0.99 (0.96)	0.98 (0.4)	0.14	0.74 (0.24)	0.0004*

Data are represented as either mean ± SD or median (IQR), *indicates a *p*-value < 0.05, and significance was tested with a Tukey HSD test or a pairwise Wilcoxon test. Body mass index (BMI), waist-hip-ratio (WHR), low-density lipoprotein (LDL), very low-density lipoprotein (VLDL), high-density lipoprotein, high-sensitivity C-reactive protein (hsCRP), baseline (BL), 3 months (3 M), 12 months (12 M)

Differences in cytokine levels at baseline between patients undergoing bariatric surgery (both RYGB and SG) and controls were analyzed using a linear model (NPX ~ case/controls) in R version 4.3.0 (IDE version 2023.21.4). This model tested if there were any significant differences in the mean cytokine levels between two groups while accounting for variability in NPX. Changes in cytokine levels after bariatric surgery were assessed using a linear mixed model with the gls procedure (NPX ~ visit + (visit|id)) in R. To investigate differential changes between RYGB and SG, an interaction term was also tested using the gls mixed model procedure in R (NPX ~ visit * surgery type + (visit|id)). In order to account for missingness and variation within participants, a linear mixed model was applied. *p*-values from cytokine analyses were False discovery rate (FDR)-corrected for multiple comparisons. An FDR-corrected *p*-value < 0.05 was considered statistically significant.

## Results

### Comparison of Clinical, Biochemical, and Anthropometric Measures at Baseline

There were no statistically significant differences in clinical characteristics, age, or sex between the RYGB and SG groups at baseline (Table 1). In accordance with the clinical indication, patients referred for bariatric surgery had higher BMIs, higher measures of glucose metabolism, were more insulin-resistant, and had higher hs-CRP levels than the controls. In the RYGB group, nine patients had T2D before the surgery and of those nine; six had both T2D and hypertension. In the SG group, nine individuals also had T2D at baseline out of those nine; five individuals also had hypertension. For an overview of anthropometric and biochemical data pooled for both surgery groups from baseline to 3 and 12 months (see supplementary Table 1).

### Plasma Cytokine Levels in Bariatric Patients vs. Controls at Baseline

**Fig. 1:**
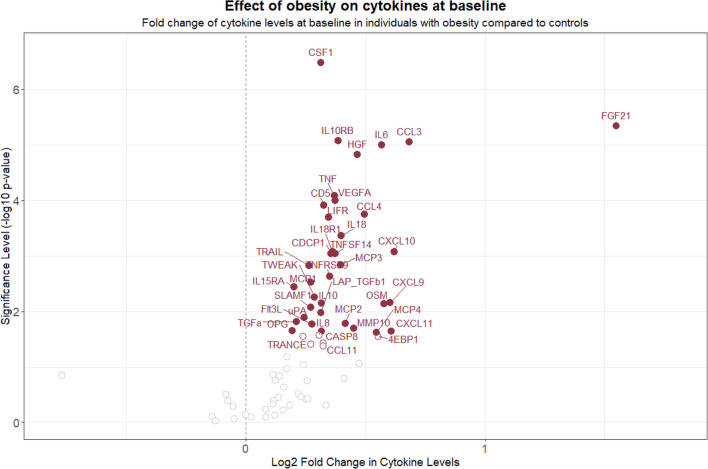
Differentially expressed cytokine levels in patients undergoing bariatric surgery (*n* = 68) compared with controls (*n* = 11) at baseline. The red unfilled dots show nominally significant differences in cytokines, the red filled dots show significant differences in cytokines after correcting for multiple testing (*p* < 5% FDR). Six cytokines were nominally higher, and 36 were significantly higher, in patients than in controls. For a comprehensive overview of the cytokines, see supplementary Table 2, where they have been ranked by *p*-value

Of the 74 cytokines examined in the study, 36 were significantly higher in bariatric patients than in controls after correcting for multiple testing (Fig. 1). The five most significantly elevated cytokines were colony-stimulating factor-1 (CSF-1), IL-6, fibroblast growth factor 21 (FGF21), interleukin-10 receptor subunit beta (IL10RB), and chemokine (C–C) ligand 3 (CCL3) (see Supplementary Table 2 for a comprehensive list of the cytokines).

### Changes in Anthropometric and Biochemical Measures 3 and 12 Months after Bariatric Surgery

In both the SG and RYGB groups, most clinical changes occurred within 3 months and persisted at 12 months, except for HDL which increased only after 12 months in both groups, and WHR, which decreased only after 12 months in the RYGB group (Table 2). In the RYGB group, nine patients had T2D at baseline, and out of those nine, six suffered from both T2D and hypertension. In the SG group, nine patients had T2D at baseline and out of those nine, five patients had hypertension. The data presented in Tables 1 and 2 contextualize the cytokine changes observed following bariatric surgery by relating them to the corresponding clinical outcomes.

### Changes in Plasma Cytokine Levels 3 and 12 Months after Bariatric Surgery

**Fig. 2:**
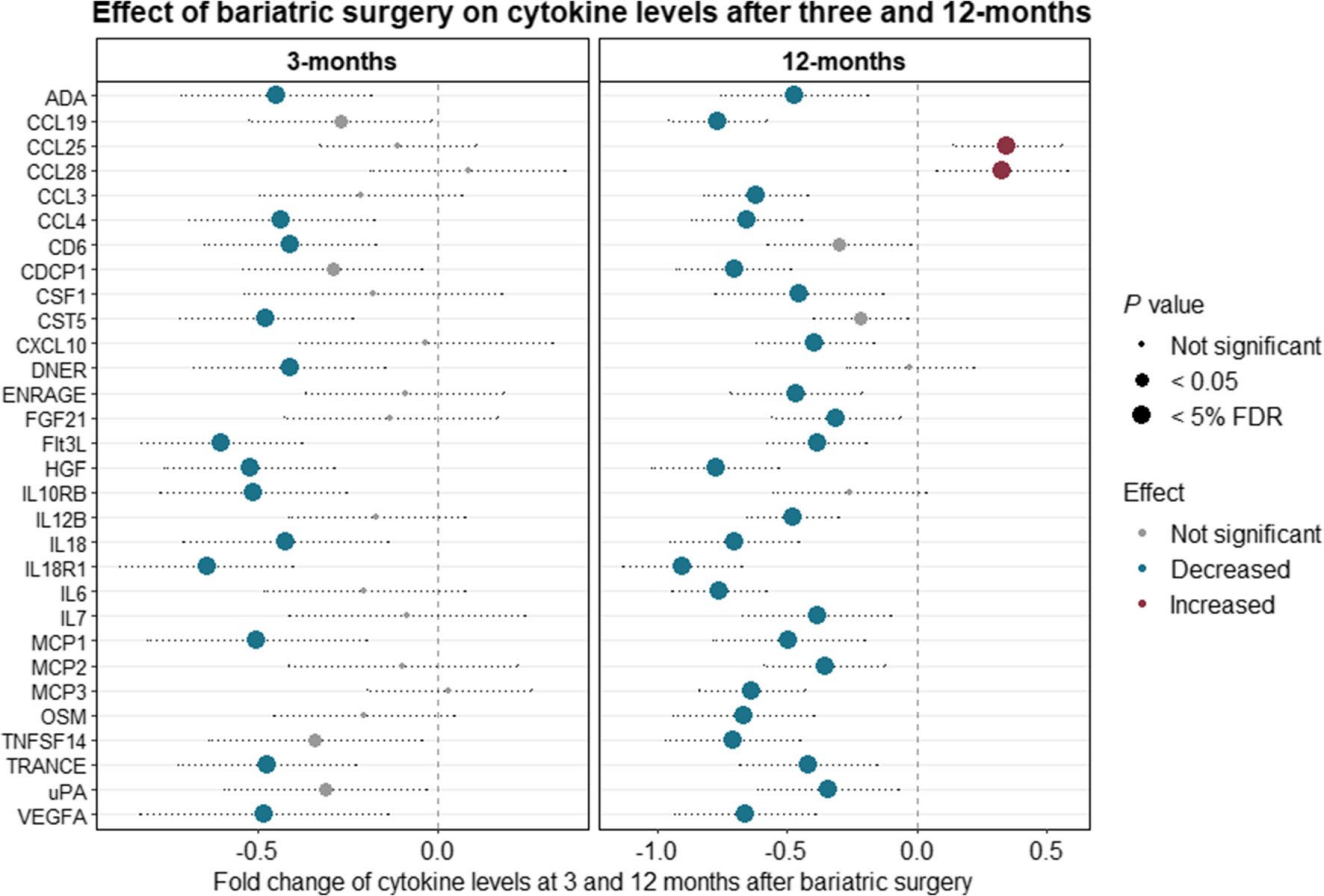
Differentially expressed cytokine levels in plasma three months and 12 months after surgery compared to baseline levels (baseline: *n* = 68, 3 months: *n* = 40, 12 months: *n* = 40). The blue dots represent significant decreases in cytokines after correcting for multiple testing (3 months: 13 cytokines had decreased significantly; 12 months: 27 cytokines had decreased significantly). The red dots represent significant increases in cytokines either after correcting for multiple testing (12 months: two cytokines had increased significantly). The medium grey dots indicate cytokines with nominally significant differences, while the small grey dots denote cytokines with non-significant differences. For a comprehensive overview of the cytokines, see supplementary Table 3–4, where they have been ranked by *p*-value

Several significant changes in plasma cytokine levels were found following bariatric surgery (Fig. 2). The plasma levels of 13 cytokines were significantly lower three months after surgery and the plasma levels of another 27 cytokines were significantly lower 12 months after surgery compared with baseline levels. Of the 13 cytokines that had decreased three months after surgery, 10 remained significantly below baseline levels after 12 months, in addition to 17 other cytokines that were found to have significantly decreased after 12 months. The 10 significantly and consistently less abundant cytokines were vascular endothelial growth factor A (VEGFA), monocyte chemoattractant protein 1 (MCP1), chemokine (C–C motif) ligand 4 (CCL4), interleukin-18 (IL-18), IL10RB (described above), interleukin-18 receptor 1 (IL18R1), TNF-related activation-induced cytokine (TRANCE), hepatocyte growth factor (HGF), FMS-related tyrosine kinase 3 ligand (Fit3L), and adenosine deaminase (ADA) (see Supplementary Tables 3 and 4 for a comprehensive list of the cytokines ranked).

No cytokines were found to be more abundant three months after surgery than at baseline. However, two cytokines, chemokine (C–C motif) ligand 25 (CCL25) and chemokine (C–C motif) ligand 28 (CCL28), had significantly increased 12 months after surgery (Fig. 2). How the changes in cytokine levels correlate with changes in clinical traits at 12 months is shown in Supplementary Fig. 2.

### Comparison of Cytokine Levels Between the RYGB and SG Groups

**Fig. 3:**
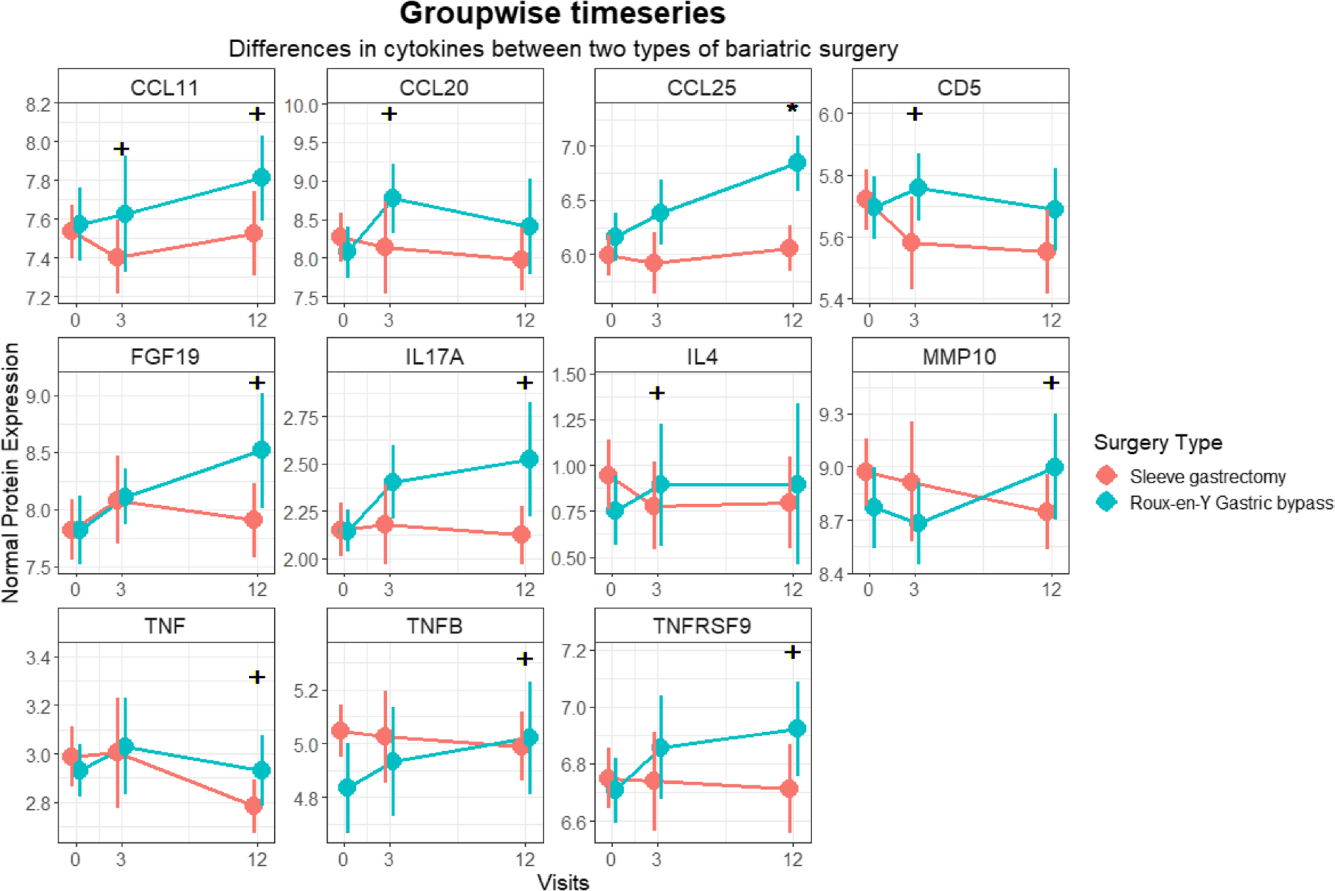
Differentially expressed cytokine levels in RYGB and SG patients at baseline, three months and 12 months after surgery. Red lines: SG, blue lines: RYGB, “ + ” indicates nominal differences between the two groups at each time point and * indicates statistically significant differences between the two groups after correcting for multiple testing. Only CCL25 is significantly different between the two groups 12 months after surgery

**Fig. 4:**
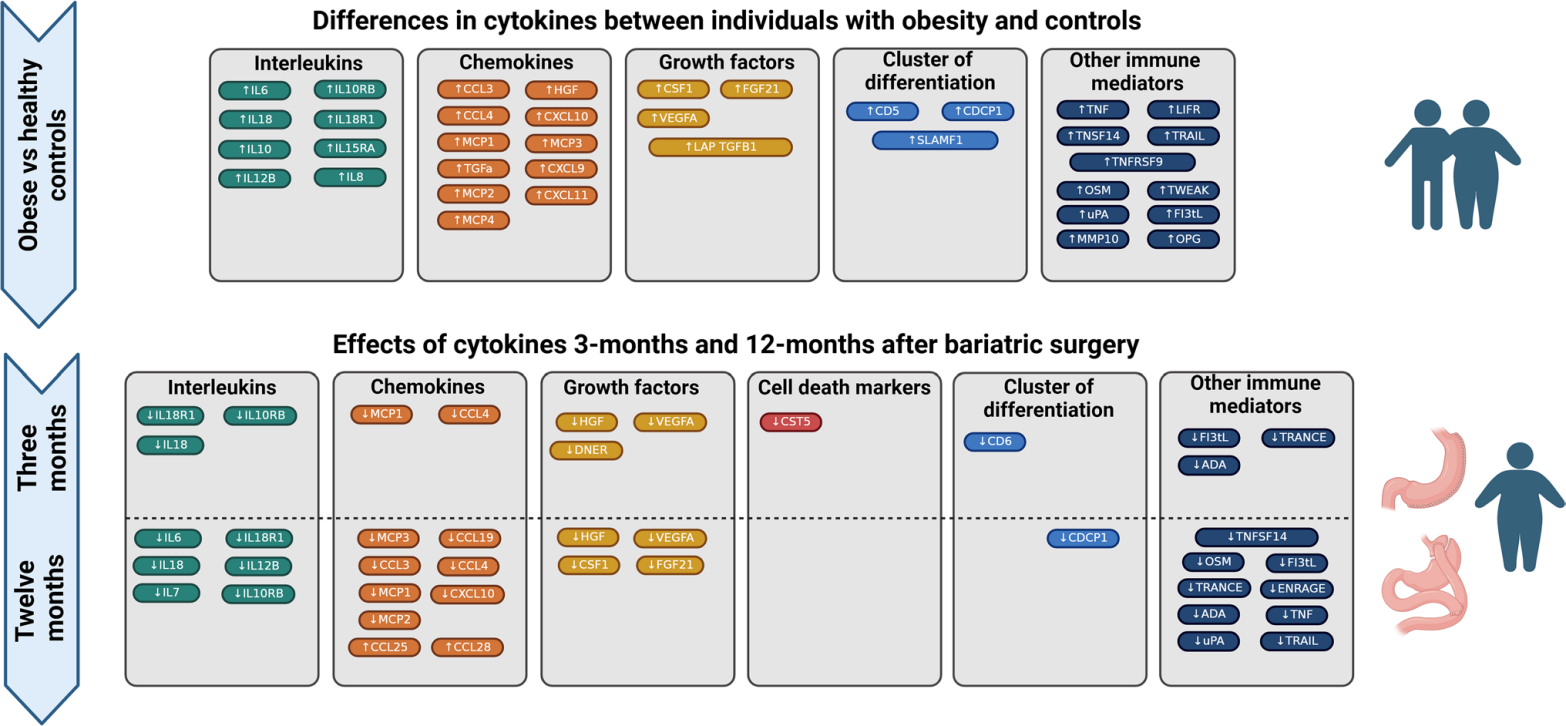
Overview of differences in plasma cytokines between individuals with obesity and controls, and in individuals with obesity three months and 12 months after bariatric surgery. Only significant cytokines have been grouped and illustrated. The significant cytokine markers were chemokines, growth factors, interleukins, cytokines associated with cell death, and related to other inflammatory processes. The inflammatory markers have been color-coded according to their primary functions, as identified in previous studies to highlight their roles in specific functional pathways. Figure 4 was created with https://www.biorender.com/

Two cytokines were nominally different at baseline (osteoprotegerin (OPG) and tumor necrosis factor B (TNFB)) (Supplementary Fig. 1); however, this association was not found to be significant after correcting for multiple comparisons. A single cytokine, CCL25, was significantly different between surgery types, having increased in the RYGB group after 12 months compared to SG at 12 months (Fig. 3). The effect of surgery on cytokines, which have been grouped into six categories, is illustrated in Fig. 4.

## Discussion

We have elucidated both the short- and long-term effects of bariatric surgery-induced weight-loss on low-grade inflammation. Our study has three main findings. First, a broad panel of plasma markers of inflammation were significantly more abundant in individuals with severe obesity compared to control subjects. Second, markers of low-grade inflammation were generally less abundant three and 12 months after bariatric surgery. Third, only minimal differences in low-grade inflammatory markers between the RYGB and SG groups were observed, with only one, CCL25, found to be significantly different between surgery types. Notably, neither the degree of weight-loss (on average about 25 kg) nor the average BMI (31.4 kg/m^2^) after 12 months differed between the two surgery groups.

The theory that obesity is linked to a low-grade, chronic inflammatory condition [3] resonates with the outcomes of this study, in which we found that 36 of 74 cytokines were elevated in individuals with severe obesity when compared to control subjects. Our finding of diminished inflammation after bariatric surgery also supports other studies of bariatric patients that examined fewer inflammatory markers [10, 14, 18]. And our findings correspond well with studies of diet-induced weight-loss, which report reduced plasma levels of inflammatory markers after a reduction in weight [19–21].

We found 13 pro-inflammatory cytokines that decreased markedly within 3 months of surgery. These changes took place alongside substantial weight-loss of an average of 19 kg and marked improvement in metabolic traits, suggesting that bariatric surgery induces a drastic and rapid improvement in low-grade inflammation.

Twelve months after bariatric surgery, the 10 cytokines that had decreased after 3 months remained significantly reduced, while the levels of a further 17 cytokines had also significantly decreased. This is suggestive of ongoing and substantial improvements in systemic, chronic, low-grade inflammation with progressive weight-loss over time.

In our study, the chemokine MCP-1 was elevated in our bariatric cohort at baseline and had decreased three and 12 months after bariatric surgery. MCP-1 is an important pro-inflammatory chemokine that is expressed by adipocytes and which has repeatedly been found to be elevated in individuals with obesity [22, 23], including in the visceral adipose tissue [24]. The presence of MCP-1 results in the recruitment and influx of monocytes into the adipose tissue. Here, these monocytes differentiate into mature macrophages that result in inflammation and augmented insulin resistance in the adipose tissue [25, 26]. The increased number of macrophages induced by the MCP-1 secretion results in increased levels of cytokines like IL-6 and TNF which contribute to both local and systemic inflammation [25, 26]. The rapid decline in MCP-1 plasma levels following surgery-induced weight loss and loss of fat mass may therefore reflect a swift and marked decrease in the number of adipose tissue macrophages [27] and an improvement in adipose tissue inflammation, which is considered the main driver of low-grade inflammation in obesity [27]. This reduction in MCP-1 levels may promote adipose tissue differentiation and decrease macrophage infiltration, thereby enhancing adipocyte function and lipid storage [25, 28]. Additionally, the concomitant decrease in macrophage-driven inflammation contributes to improved insulin sensitivity. It is likely, that MCP-1 not only serves as a pivotal connection between inflammation and obesity-related disorders like MD, CVD, and T2D [29] but also is an important driver of the subsequent reduced risks of these conditions following bariatric surgery [30, 31].

Among the interesting cytokines and chemokines that decreased substantially (Fig. 4), we found the well-known and highly pro-inflammatory cytokines IL-6, TNF, and IL-18 whose roles in adipose tissue-driven inflammation and insulin resistance are well-established [32]. Concomitantly, we also noted the reductions in VEGFA, monocyte chemo attractant protein 2 (MCP-2) and TNF-related apoptosis ligand (TRAIL), the latter of which has been reported to stimulate both MCP-1 and IL-6 expression in adipocytes [33]. Altogether, our findings point to weight-loss as being critical in reversal of chronic inflammation. As bariatric surgery provides sustained weight reduction its’ beneficial effects reflect on the life expectancy which in the Swedish Obesity Study has been reported to increase significantly by three years when compared with individuals with obesity who receives usual obesity care [30]. Probably, the reason for this is the diminished inflammation and subsequent improvement in glucose homeostasis. However, further research is needed with large-scale follow-up with in-depth characterization of the inflammatory profile.

Given the considerable anatomical and physiological discrepancies between RYGB and SG, it is notable that our results point towards a largely similar impact on low-grade inflammation, with no differences observed in the cytokines that decreased after surgery. After SG, the only change is a longitudinal excision of the major curvature of the stomach. By contrast, after RYGB, there is an extensive rearrangement of the upper gastrointestinal tract and bypassing of a large part of the jejunum. With this in mind, we found two cytokines, CCL28 and CCL25 that had increased 12 months after surgery. CCL28 is a regulator of cellular chemotaxis, and CCL25 has been reported to be inversely associated with adiposity [34]. We observed an increase of CCL28 with weight reduction, and our combined analysis of both surgery groups showed that CCL25 had significantly increased 12 months after surgery; however, our interaction analysis revealed that this increase was mostly driven by the RYGB group, while the change in SG was non-significant. Thus, CCL25 was the only cytokine that differed between RYGB and SG groups 12 months after surgery.

CCL25 is a CCR9 (G-coupled protein receptor) ligand and is believed to play a role in the differentiation of T-cells [35]. Recent data suggest that human pancreatic beta cell function is impaired through the binding of CCL25 to CCR9, which hinders insulin secretion and exacerbates metabolic MD [35]. As such, our finding of increased CCL25 would seem to contradict the assumption that RYGB improves T2D [36]. As CCL25 expression occurs in the thymus and intestinal cells [37], it could be speculated that the CCL25 increase following RYGB simply mirrors the upsurge in absorptive intestinal area, which is well-established as taking place following RYGB [38]. Because CCR9 was not a part of our inflammatory panel, we cannot know if the receptor was concomitantly upregulated. In addition, we have found no weight-loss studies or bariatric studies with specific mention of CCL25. Consequently, it remains elusive how our finding of increased CCL25 expression in RYGB should be interpreted clinically and further research is needed.

That only CCL25 differed between the RYGB and SG groups appears remarkable. Yet this result is in accordance with the comparable weight-loss and improvements in glucose homeostasis (but not lipid levels) that we observed in both surgery groups. This indicates that it is probably the weight-loss itself, and not the type of surgery, that reduces inflammation. In addition, our finding supports existing data that SG and RYGB have a very similar impact on the components of the metabolic syndrome, as well as MASLD [39, 40].

There are some limitations to our study. In Denmark, patients referred for RYGB and SG must meet the clinical criteria issued by the Danish Health Authorities, which typically mandate a diet-induced weight loss of 8% before a bariatric procedure can be performed. As our participants’ baseline blood samples were drawn after this weight loss, we may have missed some of the effects of the weight-loss on inflammation. The participants were also undergoing a non-randomized type of surgery. It has been established that the peak weight-loss following RYGB occurs within 1 to 1.5 years [41]. Moreover, there is evidence that approximately 2 years are necessary for stabilization of the inflammatory profile [41], suggesting that a follow-up period longer than 12 months is probably needed to show the full impact of bariatric surgery.

A strength of this study is its comprehensive cytokine profiling using the Olink platform, which can measure low-abundance proteins such as cytokines. Another strength is that our study has a similar or longer follow-up than previous studies in the field and provides new insights into the longer-term impacts of weight-loss on the cytokine profile following bariatric surgery.

In conclusion, we show that low-grade inflammation rapidly and continuously improves following RYGB and SG and that the effects on the cytokine profile of these modes of surgery are remarkably similar.

## Supplementary Information

Below is the link to the electronic supplementary material.

Supplementary file1 (PDF 737 KB)

## Data Availability

The data that support the findings of this study are not openly available due to reasons of sensitivity and are available from the corresponding author upon reasonable request. Data are located in controlled access data storage at University of Copenhagen and Hvidovre University Hospital.

## Abbreviations

T2D: Type 2 diabetes ()
RYGB: Roux-en-Y gastric bypass
SG: Sleeve gastrectomy
MD: Metabolic dysfunction
IL-6: Interleukin-6
BMI: Body mass index
NPX: Normalized protein expression
ANOVA: A two-way analysis of variance
HOMA-IR: Homeostasis model assessment of insulin resistance
WHR: Waist-hip ratio
HDL: High-density lipoprotein
HbA1c: Hemoglobin A1c
LDL: Low-density lipoprotein
CVD: Cardiovascular disease
NAS: NAFLD activity score
CSF-1: Colony-stimulating factor-1
FGF21: Fibroblast growth factor 21
IL10RB: Interleukin-10 receptor subunit beta
CCL3: Chemokine (C–C) ligand 3
VEGFA: Vascular endothelial growth factor A
MCP1: Monocyte chemoattractant protein 1
MCP2: Monocyte chemoattractant protein 2
CCL4: Chemokine (C–C motif) ligand 4
IL-18: Interleukin-18
IL18R1: Interleukin-18 receptor 1
TRANCE: TNF-related activation-induced cytokine
HGF: Hepatocyte growth factor
Fit3L: FMS-related tyrosine kinase 3 ligand
ADA: Adenosine deaminase
CCL25: Chemokine (C–C motif) ligand 25
CCL28: Chemokine (C–C motif) ligand 28
OPG: Osteoprotegerin
TNFB: Tumor necrosis factor B
CCL11: Chemokine (C–C motif) 11
CCL20: Chemokine (C–C motif) 20
CD5: Cluster of differentiation 5
IL-17A: Interleukin-17A
IL-4: Interleukin-4
MMP10: Matrix metalloproteinase-10
TNFRSF9: Tumor necrosis factor receptor superfamily 9
MASLD: Metabolic dysfunction-associated steatotic liver disease
FDR: False discovery rate
TRAIL: TNF-related apoptosis ligand
