# Supplementary material for: Changes in Inflammatory Markers Following Bariatric Surgery and the Impact of the Surgical Procedure: A 12-Month Longitudinal Study
Source: Obes Surg. 2025 May 27;35(7):2626–37. doi: 10.1007/s11695-024-07629-z (PMC12271296; doi:10.1007/s11695-024-07629-z)
Supplement: Supplementary file 1 — Supplementary file1 (PDF 737 KB) [file 11695_2024_7629_MOESM1_ESM.pdf]

Supplementary Tables and Figures

***Supplementary Table 1** – Changes in anthropometric and biochemical characteristics after bariatric surgery.*

	Baseline	3-months	12-months	*P*-value
n	*n=40*	*n=40*	*n=40*	
**Weight (kg)**	125.5 ± 19.19	107.04 ± 17.78	93.88 ± 16.36	8.33e-15*
**BMI (kg/m^2)**	41.94 ± 5.78	35.85 ± 5.53	31.43 ± 5.05	0.0008 *
**Waist circumference (cm)**	121.0 ± 11.2	105.93 ± 12.1	97.31 ± 12.48	0.0002 *
**Hip circumference (cm)**	131 (23)	119.5 (20)	112.39 (18.63)	9.47e-12 *
**WHR**	0.92 ± 0.12	0.89 ± 0.11	0.87 ± 0.1	0.066
**Glucose (mmol/L)**	6.1 (1.1)	5.4 (0.5)	5.4 (0.43)	9.71e-08*
**Insulin (pmol/L)**	128 (70.5)	71 (48)	60 (36.5)	6.33e-11*
**C-peptide (pmol/L)**	1180 (350)	888 (332)	778 (284)	4.95e-11*
**HbA1c (mmol/mol)**	37 (5)	36 (5)	33.5 (5)	0.11
**HOMA-IR**	4.9 (3.2)	2.33 (1.7)	2.04 (1.07)	3.5e-13*
**hsCRP (mg/L)**	4.2 (5.2)	2.3 (3.8)	0.8 (1.1)	1.67e-10
**Total cholesterol (mmol/L)**	4.37 ± 0.9	3.89 ± 1.0	4.04 ± 1.3	0.09
**LDL (mmol/L)**	2.51 ± 0.84	2.22 ± 0.88	2.23 ± 1.02	0.24
**VLDL (mmol/L)**	0.6 (0.33)	0.4 (0.1)	0.4 (0.2)	3.59e-06
**HDL (mmol/L)**	1.18 ± 0.3	1.18 ± 0.27	1.47 ± 0.32	4.28e-08*
**Triglycerides (mmol/L)**	1.46 ± 0.64	1.11 ± 0.48	0.96 ± 0.54	0.0002*

*Supplementary table 1 – Data are represented as either mean ± SD or median (IQR), * indicates a p-value < 0.05 and significance was tested with a two-way ANOVA or Kruskal-Wallis test. Body mass index (BMI), Low-density lipoprotein (LDL), Very low-density lipoprotein (VLDL), High-density lipoprotein (HDL), High-sensitivity C-reactive protein (hsCRP), Waist-hip ratio (WHR).*

Supplementary Table 2 - *Controls vs. patients with obesity referred for bariatric surgery at baseline. Cytokines are ranked from the highest in significance to the lowest.*

Assay	std.error	p.value	CI	adjp
**CSF1**	0.28	3.28E-07	1.02:2.15	2.43E-05
**IL6**	0.31	1.01E-05	0.84:2.06	0.000149
**FGF21**	0.31	4.61E-06	0.9:2.13	0.000149
**IL10RB**	0.29	8.53E-06	0.82:1.99	0.000149
**CCL3**	0.29	8.84E-06	0.79:1.93	0.000149
**HGF**	0.28	1.48E-05	0.74:1.86	0.000183
**TNF**	0.3	8.31E-05	0.65:1.85	0.000879
**VEGFA**	0.27	0.000101	0.58:1.66	0.00093
**CD5**	0.3	0.000122	0.61:1.8	0.001001
**CCL4**	0.28	0.000179	0.54:1.64	0.001323
**LIFR**	0.29	0.0002	0.55:1.71	0.001344
**IL18**	0.28	0.000439	0.47:1.57	0.002709
**CDCP1**	0.3	0.000914	0.44:1.65	0.004278
**TNFSF14**	0.27	0.000925	0.39:1.45	0.004278
**IL18R1**	0.3	0.000844	0.45:1.65	0.004278
**CXCL10**	0.31	0.000849	0.46:1.7	0.004278
**MCP3**	0.32	0.001454	0.42:1.71	0.00613
**TRAIL**	0.32	0.001491	0.42:1.7	0.00613
**TNFRSF9**	0.29	0.002371	0.34:1.5	0.009234
**MCP1**	0.31	0.002969	0.34:1.59	0.010986
**IL15RA**	0.31	0.003698	0.31:1.54	0.013031
**TWEAK**	0.3	0.005631	0.26:1.46	0.018941
**CXCL9**	0.31	0.006948	0.24:1.49	0.021748
**OSM**	0.33	0.007347	0.25:1.55	0.021748
**IL10**	0.31	0.007218	0.24:1.47	0.021748
**SLAMF1**	0.29	0.008503	0.21:1.37	0.0242
**LAP_TGFb1**	0.3	0.010606	0.19:1.38	0.029067
**uPA**	0.32	0.01298	0.18:1.47	0.034305
**TGFa**	0.3	0.015479	0.15:1.36	0.039498
**MCP2**	0.31	0.016502	0.14:1.39	0.040705
**Flt3L**	0.31	0.017159	0.14:1.37	0.04096
**MMP10**	0.32	0.020109	0.12:1.41	0.046501

**OPG**	0.29	0.022432	0.1:1.27	0.048518
**CXCL11**	0.31	0.022948	0.1:1.33	0.048518
**IL12B**	0.32	0.022672	0.11:1.4	0.048518
**MCP4**	0.32	0.024232	0.1:1.37	0.049811
**IL8**	0.31	0.026937	0.08:1.33	0.053874
**CCL23**	0.28	0.028478	0.07:1.18	0.054046
**4EBP1**	0.33	0.028483	0.08:1.41	0.054046
**CASP8**	0.31	0.037753	0.04:1.29	0.069844
**TRANCE**	0.33	0.039817	0.03:1.34	0.071865
**CCL11**	0.3	0.043014	0.02:1.22	0.075786
**CCL19**	0.3	0.066731	-0.04:1.14	0.114839
**CCL20**	0.3	0.086786	-0.08:1.13	0.145958
**CX3CL1**	0.32	0.092191	-0.09:1.2	0.151603
**CD244**	0.29	0.108536	-0.11:1.06	0.174602
**PDL1**	0.32	0.138397	-0.16:1.13	0.217901
**CXCL5**	0.31	0.14301	-1.09:0.16	0.220474
**CD6**	0.32	0.148299	-0.17:1.1	0.223961
**CCL25**	0.3	0.160126	-0.17:1.02	0.236987
**ADA**	0.29	0.174618	-0.18:0.99	0.253367
**IFNg**	0.32	0.179114	-0.2:1.07	0.254893
**IL7**	0.28	0.234927	-0.22:0.89	0.328011
**CXCL6**	0.31	0.310613	-0.3:0.92	0.425655
**CCL28**	0.32	0.319132	-0.97:0.32	0.429378
**IL17C**	0.31	0.350833	-0.32:0.9	0.459298
**TNFB**	0.33	0.353784	-0.35:0.97	0.459298
**SIRT2**	0.31	0.381385	-0.34:0.88	0.478347
**STAMBP**	0.31	0.38012	-0.34:0.88	0.478347
**CD40**	0.3	0.400805	-0.35:0.86	0.494326
**FGF19**	0.31	0.416199	-0.88:0.37	0.504898
**SCF**	0.34	0.468739	-0.43:0.93	0.559463
**MMP1**	0.33	0.492328	-0.43:0.89	0.572093
**NT3**	0.32	0.494783	-0.42:0.85	0.572093
**DNER**	0.33	0.525024	-0.88:0.45	0.597719
**CST5**	0.32	0.586797	-0.47:0.82	0.657924
**CD8A**	0.33	0.605583	-0.49:0.84	0.668853

**IL4**	0.3	0.735368	-0.5:0.7	0.800254
**IL17A**	0.3	0.756515	-0.51:0.7	0.811335
**AXIN1**	0.31	0.806344	-0.54:0.69	0.828742
**CXCL1**	0.32	0.798842	-0.71:0.55	0.828742
**IL10RA**	0.31	0.805385	-0.7:0.55	0.828742
**ST1A1**	0.35	0.873687	-0.75:0.64	0.885656
**ENRAGE**	0.34	0.941072	-0.65:0.7	0.941072

Supplementary Table 2 - shows each cytokine in the Assay column after being run through a linear mixed model, with an estimate, standard error (std.error), p*-value (p.value, not adjusted for multiple testing), 95% confidence interval (CI), and a* p*-value adjusted for multiple testing (adjp).*

Supplementary Table 3 *- Estimated changes in plasma cytokine levels (mean differences) from baseline to three months after bariatric surgery.*

Assay	estimate	std.error	p.value	CI	adjp
**IL18R1**	-0.67	0.12	1.77E-07	-0.91:-0.43	9.44E-06
**Flt3L**	-0.62	0.11	2.55E-07	-0.84:-0.39	9.44E-06
**HGF**	-0.56	0.12	5.76E-06	-0.8:-0.33	0.000142
**IL10RB**	-0.54	0.13	6.42E-05	-0.8:-0.29	0.001188
**TRANCE**	-0.51	0.13	0.000108	-0.76:-0.26	0.0016
**CST5**	-0.48	0.12	0.000183	-0.72:-0.23	0.00226
**MCP1**	-0.55	0.16	0.00058	-0.85:-0.24	0.005365
**CCL4**	-0.47	0.13	0.000517	-0.72:-0.21	0.005365
**CD6**	-0.42	0.12	0.000744	-0.66:-0.18	0.006119
**ADA**	-0.46	0.14	0.000875	-0.72:-0.19	0.006477
**VEGFA**	-0.59	0.17	0.000972	-0.93:-0.25	0.006536
**IL18**	-0.46	0.15	0.001968	-0.74:-0.17	0.012138
**DNER**	-0.39	0.14	0.004944	-0.65:-0.12	0.028145
**uPA**	-0.37	0.14	0.011578	-0.65:-0.09	0.059508
**SLAMF1**	-0.35	0.14	0.012062	-0.63:-0.08	0.059508
**OPG**	-0.37	0.15	0.015335	-0.66:-0.07	0.066814
**TNFSF14**	-0.37	0.15	0.015349	-0.66:-0.07	0.066814
**CDCP1**	-0.3	0.13	0.020883	-0.55:-0.05	0.085852
**IL6**	-0.31	0.14	0.025492	-0.58:-0.04	0.099285
**CSF1**	-0.39	0.18	0.027145	-0.74:-0.05	0.100435
**CCL19**	-0.28	0.13	0.028799	-0.54:-0.03	0.101481

**TRAIL**	-0.34	0.16	0.035664	-0.65:-0.03	0.11996
**OSM**	-0.27	0.13	0.039225	-0.52:-0.02	0.126201
**TGFa**	-0.36	0.17	0.04163	-0.7:-0.02	0.128361
**CCL3**	-0.28	0.14	0.055961	-0.56:0	0.159274
**FGF19**	0.31	0.16	0.054219	0:0.63	0.159274
**IFNg**	-0.29	0.16	0.064106	-0.59:0.01	0.175698
**CCL23**	0.31	0.17	0.081772	-0.04:0.65	0.216113
**FGF21**	-0.26	0.15	0.090111	-0.55:0.04	0.22823
**IL12B**	-0.21	0.13	0.092526	-0.46:0.03	0.22823
**IL17C**	0.23	0.15	0.134695	-0.07:0.53	0.314009
**IL17A**	0.29	0.19	0.135787	-0.09:0.67	0.314009
**IL10RA**	-0.17	0.13	0.19903	-0.42:0.09	0.446309
**CCL11**	-0.17	0.13	0.211407	-0.43:0.09	0.460121
**CD8A**	0.13	0.11	0.259128	-0.09:0.34	0.547006
**AXIN1**	0.18	0.17	0.280895	-0.14:0.5	0.547006
**IL15RA**	-0.17	0.15	0.273075	-0.47:0.13	0.547006
**MMP10**	-0.19	0.18	0.280602	-0.54:0.16	0.547006
**IL4**	-0.14	0.14	0.298086	-0.41:0.13	0.565599
**LIFR**	-0.15	0.15	0.308641	-0.44:0.14	0.570985
**NT3**	0.19	0.19	0.317194	-0.18:0.56	0.572497
**CCL25**	-0.11	0.11	0.340832	-0.32:0.11	0.600514
**MMP1**	0.13	0.14	0.353191	-0.14:0.4	0.607817
**CD5**	-0.12	0.13	0.385186	-0.38:0.14	0.615155
**4EBP1**	-0.14	0.16	0.390706	-0.45:0.18	0.615155
**CD40**	0.16	0.18	0.372708	-0.19:0.51	0.615155
**TNFRSF9**	0.14	0.16	0.379326	-0.17:0.45	0.615155
**MCP2**	-0.13	0.16	0.41451	-0.45:0.18	0.638463
**CCL20**	0.15	0.19	0.422766	-0.22:0.52	0.638463
**CX3CL1**	-0.11	0.14	0.447109	-0.39:0.17	0.661721
**CXCL10**	-0.13	0.18	0.462021	-0.48:0.22	0.670383
**IL8**	-0.11	0.16	0.506348	-0.42:0.21	0.671058
**IL7**	-0.12	0.17	0.494283	-0.45:0.21	0.671058
**CCL28**	0.09	0.14	0.511149	-0.18:0.36	0.671058
**ENRAGE**	-0.1	0.14	0.491414	-0.37:0.18	0.671058
**CASP8**	-0.11	0.17	0.519651	-0.44:0.22	0.671058

**TWEAK**	-0.12	0.18	0.500935	-0.48:0.23	0.671058
**STAMBP**	0.11	0.17	0.525964	-0.23:0.44	0.671058
**CXCL9**	0.11	0.19	0.564744	-0.27:0.49	0.708323
**IL10**	-0.12	0.23	0.607358	-0.57:0.33	0.736795
**ST1A1**	0.09	0.16	0.601629	-0.24:0.41	0.736795
**SIRT2**	0.07	0.17	0.689224	-0.26:0.39	0.822622
**CXCL6**	-0.05	0.14	0.751246	-0.32:0.23	0.882416
**LAP_TGFb1**	-0.04	0.18	0.810601	-0.39:0.3	0.937257
**MCP3**	-0.02	0.12	0.880622	-0.25:0.21	0.965263
**CXCL11**	0.03	0.18	0.886998	-0.33:0.38	0.965263
**PDL1**	-0.02	0.17	0.883903	-0.35:0.3	0.965263
**CXCL5**	0.02	0.15	0.886325	-0.28:0.32	0.965263
**CD244**	0.02	0.16	0.91877	-0.3:0.34	0.971271
**MCP4**	0.01	0.13	0.916866	-0.24:0.27	0.971271
**TNF**	0.01	0.19	0.944891	-0.36:0.39	0.984816
**CXCL1**	0.01	0.16	0.973748	-0.31:0.32	0.99488
**SCF**	0	0.13	0.981435	-0.25:0.26	0.99488
**TNFB**	0	0.18	0.998198	-0.35:0.35	0.998198

Supplementary Table 3 - shows each cytokine in the Assay column after being run through a linear mixed model, with an estimate, standard error (std.error), p*-value (p.value, not adjusted for multiple testing), 95% confidence interval (CI), and a* p*-value adjusted for multiple testing (adjp).*

Supplementary Table 4 - *Estimated changes in plasma cytokine levels (mean differences) from baseline to 12 months after bariatric surgery.*

**Assay**	**estimate**	**std.error**	**p.value**	**CI**	**adjp**
**IL6**	-0.85	0.09	1.91E-16	-1.03:-0.67	1.41E-14
**CCL19**	-0.78	0.1	3.55E-13	-0.97:-0.59	8.76E-12
**IL18R1**	-0.95	0.12	2.90E-13	-1.18:-0.72	8.76E-12
**CDCP1**	-0.74	0.11	6.30E-10	-0.96:-0.52	1.17E-08
**MCP3**	-0.69	0.11	1.40E-09	-0.89:-0.48	1.73E-08
**HGF**	-0.82	0.13	1.24E-09	-1.07:-0.57	1.73E-08
**CCL3**	-0.65	0.1	2.97E-09	-0.85:-0.45	3.14E-08
**CCL4**	-0.67	0.11	7.85E-09	-0.88:-0.45	7.26E-08
**IL18**	-0.74	0.13	3.46E-08	-0.99:-0.49	2.84E-07
**TNFSF14**	-0.74	0.13	1.56E-07	-1:-0.48	1.15E-06
**IL12B**	-0.5	0.09	1.76E-07	-0.67:-0.32	1.19E-06
**OSM**	-0.75	0.14	3.23E-07	-1.02:-0.47	1.99E-06
**VEGFA**	-0.72	0.14	6.43E-07	-0.99:-0.45	3.66E-06
**Flt3L**	-0.4	0.1	5.81E-05	-0.6:-0.21	0.000307
**MCP1**	-0.59	0.14	8.01E-05	-0.87:-0.3	0.000395
**CSF1**	-0.61	0.16	0.000286	-0.93:-0.29	0.001323
**CXCL10**	-0.42	0.12	0.000354	-0.65:-0.2	0.001543
**ENRAGE**	-0.47	0.13	0.000417	-0.73:-0.22	0.001713
**TRANCE**	-0.46	0.14	0.00095	-0.73:-0.19	0.003623
**ADA**	-0.49	0.15	0.000979	-0.77:-0.2	0.003623
**MCP2**	-0.4	0.12	0.001198	-0.63:-0.16	0.004223
**FGF21**	-0.4	0.13	0.002042	-0.64:-0.15	0.006626
**CCL25**	0.34	0.11	0.002059	0.13:0.55	0.006626
**TNF**	-0.36	0.13	0.006954	-0.61:-0.1	0.021443
**IL7**	-0.4	0.15	0.007814	-0.68:-0.11	0.022239
**uPA**	-0.38	0.14	0.007705	-0.66:-0.1	0.022239
**CCL28**	0.34	0.13	0.009549	0.09:0.6	0.026172
**TRAIL**	-0.36	0.14	0.012668	-0.63:-0.08	0.032325
**IL10RB**	-0.38	0.15	0.012486	-0.67:-0.09	0.032325
**CD6**	-0.33	0.14	0.020615	-0.62:-0.05	0.05085
**CST5**	-0.22	0.09	0.02137	-0.4:-0.03	0.051011
**IL17A**	0.35	0.16	0.028165	0.04:0.67	0.065132

**OPG**	-0.3	0.14	0.032465	-0.58:-0.03	0.0728
**4EBP1**	-0.39	0.19	0.039877	-0.75:-0.02	0.086792
**FGF19**	0.37	0.18	0.044103	0.01:0.72	0.093246
**PDL1**	-0.32	0.16	0.04693	-0.64:-0.01	0.094921
**CD5**	-0.28	0.14	0.047461	-0.56:-0.01	0.094921
**SCF**	0.3	0.16	0.05919	-0.01:0.61	0.115265
**CXCL11**	-0.27	0.14	0.062075	-0.55:0.01	0.117783
**CASP8**	-0.35	0.19	0.06484	-0.72:0.02	0.119954
**IL8**	-0.25	0.15	0.10095	-0.55:0.05	0.182202
**TNFRSF9**	0.22	0.14	0.110177	-0.05:0.49	0.194122
**IL15RA**	-0.19	0.12	0.122086	-0.43:0.05	0.205326
**IL10**	-0.22	0.14	0.120186	-0.5:0.06	0.205326
**CD244**	-0.24	0.16	0.144703	-0.55:0.08	0.223857
**LIFR**	0.23	0.16	0.145204	-0.08:0.55	0.223857
**CXCL6**	-0.21	0.14	0.139778	-0.49:0.07	0.223857
**SIRT2**	-0.26	0.17	0.139137	-0.6:0.08	0.223857
**MCP4**	-0.18	0.13	0.174808	-0.43:0.08	0.263996
**LAP_TGFb1**	-0.22	0.16	0.184758	-0.54:0.1	0.270977
**MMP10**	-0.16	0.12	0.186754	-0.4:0.08	0.270977
**CXCL5**	-0.23	0.18	0.205743	-0.58:0.12	0.292788
**IL10RA**	-0.19	0.15	0.21274	-0.48:0.1	0.297033
**SLAMF1**	-0.15	0.12	0.22427	-0.4:0.09	0.299527
**IFNg**	-0.16	0.13	0.220533	-0.41:0.09	0.299527
**STAMBP**	-0.22	0.18	0.226669	-0.56:0.13	0.299527
**IL4**	-0.2	0.17	0.231587	-0.54:0.13	0.300657
**CX3CL1**	0.17	0.15	0.256091	-0.13:0.47	0.326737
**IL17C**	0.13	0.13	0.305851	-0.12:0.39	0.38361
**CCL11**	0.12	0.13	0.332875	-0.12:0.37	0.410546
**CXCL1**	-0.15	0.18	0.38555	-0.5:0.19	0.460172
**TGFa**	-0.15	0.17	0.381005	-0.48:0.18	0.460172
**CD40**	-0.13	0.15	0.40506	-0.43:0.17	0.475784
**CCL23**	-0.11	0.16	0.484088	-0.42:0.2	0.559727
**AXIN1**	-0.12	0.17	0.491932	-0.46:0.22	0.560045
**TWEAK**	-0.07	0.16	0.675278	-0.38:0.25	0.745829
**CCL20**	-0.08	0.19	0.674624	-0.45:0.29	0.745829

**ST1A1**	-0.05	0.16	0.764137	-0.36:0.27	0.831561
**NT3**	0.05	0.18	0.778908	-0.3:0.4	0.835351
**CD8A**	0.03	0.1	0.800802	-0.17:0.22	0.846562
**CXCL9**	-0.03	0.14	0.839957	-0.31:0.25	0.875448
**MMP1**	0	0.14	0.973351	-0.27:0.26	0.973351
**DNER**	0	0.13	0.96901	-0.25:0.24	0.973351
**TNFB**	0.01	0.13	0.951599	-0.25:0.27	0.973351

Supplementary Table 4 - shows each cytokine in the Assay column after being run through a linear mixed model, with an estimate, standard error (std.error), p-value (p.value, not adjusted for multiple testing), 95% confidence interval (CI), and a p-value adjusted for multiple testing (adjp).

***Supplementary Figure 1 –** Two cytokines that were nominally different at baseline between the two groups of patients referred for bariatric surgery.*

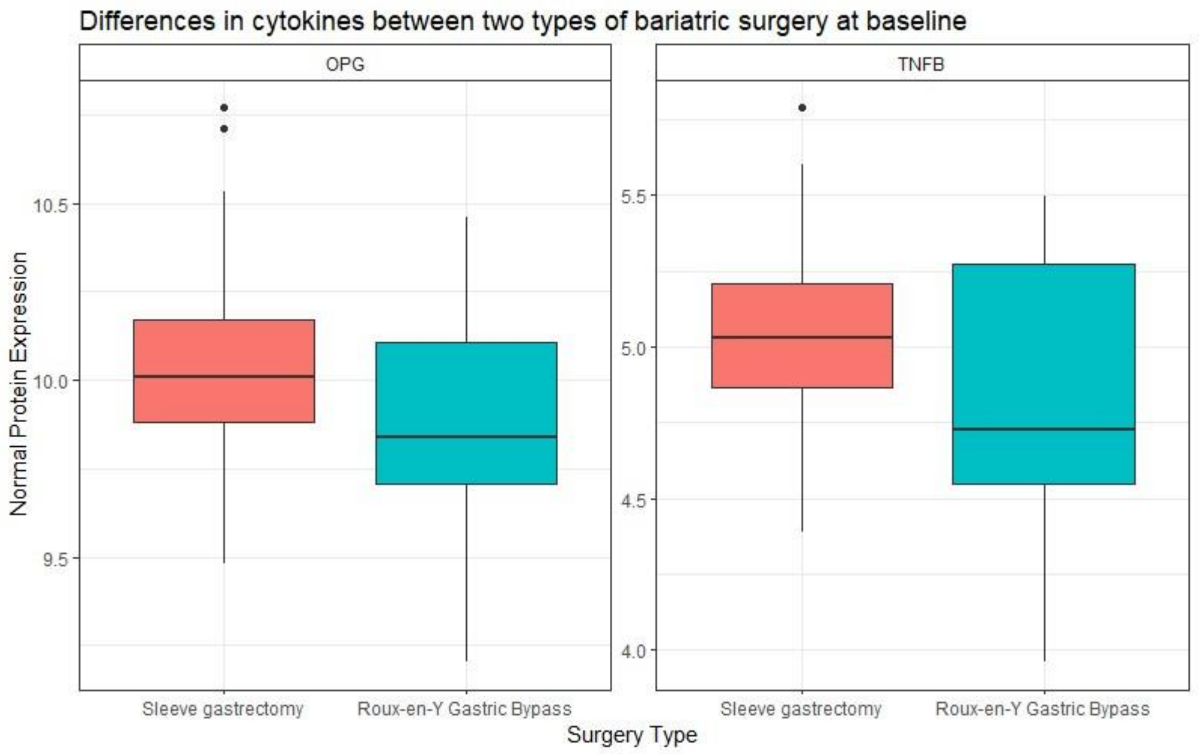

Supplementary Figure 1 - Boxplots displaying the baseline differences in cytokine levels between two types of bariatric surgery: sleeve gastrectomy (red) and Roux-en-Y gastric bypass (blue). The panels represent normalized protein expression levels for OPG (left) and TNFB (right). Black horizontal lines within the boxes indicate median values, and the box limits represent the interquartile range (IQR). Whiskers extend to 1.5 times the IQR, and points beyond the whiskers are outliers.

***Supplementary Figure 2** – Correlation heatmap between delta changes in cytokines and clinical traits at 12 months post-surgery with 14 significant correlations.*

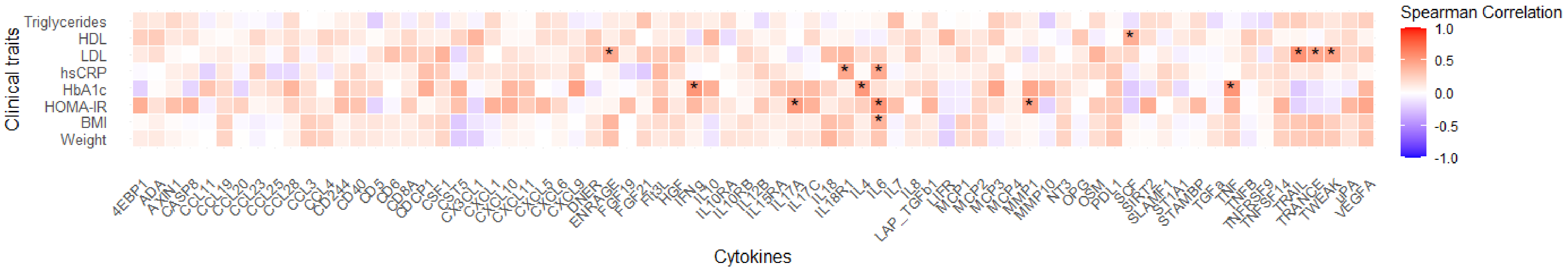

Supplementary Figure 2 - The heatmap illustrates Spearman correlation coefficients between cytokines (x-axis) and clinical traits (y-axis) at 12 months following bariatric surgery. Positive correlations are shown in shades of red, while negative correlations are shown in shades of blue, with intensity reflecting the strength of the correlation. Black asterisks (*) indicate statistically significant correlations adjusted for multiple testing correction (p < 0.05).
